# Foam Polymers in Multifunctional Insulating Coatings

**DOI:** 10.3390/polym13213698

**Published:** 2021-10-27

**Authors:** Ter-Zakaryan K. A., Zhukov A. D., Bobrova E. Yu., Bessonov I. V., Mednikova E. A.

**Affiliations:** 1 OOO TEPOFOL, Shcherbakovskaya St., 3, 105318 Moscow, Russia; 2 National Research Moscow State University of Civil Engineering (NRU MGSU), Yaroslavl Highway, 26, 129337 Moscow, Russia; lj211@yandex.ru; 3 Institute of Construction and Housing and Communal Services [GASIS] of National Research University Higher School of Economics (NRU HSE), Pokrovsky Boulevard, 109028 Moscow, Russia; mla-gasis@mail.ru; 4 Research Institute of Building Physics, Russian Academy of Architecture and Building Sciences, Locomotive Passage, 21, 127238 Moscow, Russia; bessonoviv@mail.ru; 5 The Centre of Architecture and Design at the Institute of Construction and Housing and Communal Services [GASIS] of National Research University Higher School of Economics (NRU HSE), Pokrovsky Boulevard, 11, 109028 Moscow, Russia; lisamednikova97@gmail.com

**Keywords:** polyethylene foam, insulating coating, locking joint, thermal resistance, framed building, frameless building, snow conservation

## Abstract

The application of foamed polymers as one of the components of insulating coatings allows to solve the problems of energy saving and creation of optimal operating conditions for constructions. The systems of application of energy-efficient heat-insulating materials must consider both the particularities of the insulating materials and the functional orientation of the constructions. The implementation of the concept of seamless insulating coatings implies the achievement of thermal effect and reduction in air permeability both by means of the application of thermal insulation with low thermal conductivity and the minimization of junctions between separate elements of the insulating coating, which is achieved using elastic foamed polymers and, first of all, polyethylene foam. Construction of seamless insulating coatings creates practically impermeable heat, vapor, and water barriers along the outer perimeter of the insulated object. Multilayer products based on polyethylene foam represent a relatively new material—a fact that requires examination of their properties, as well as under various operating conditions, and development of a methodology for evaluation of the operational resistance of these materials in structures of different purposes, including cold conservation. The performed tests have shown that the compressive strength at 10% deformation is determined by the function of load application area and varies from 70 kPa during the test of cube samples of 10 × 10 × 10 in size to 260 kPa for areas exceeding 100 m^2^. The longitudinal tensile strength amounts to 80–92 kPa, and the strength of the weld seam is equal to 29–32 kPa. It has been established that the values of thermal conductivity of polyethylene foam with an average density of 18–20 kg/m^3^ amounts to 0.032–0.034 W/(m·K), diffusion moisture absorption is equal to 0.44 kg/m^2^ without a metallized coating and 0.37 kg/m^2^ with a metallized coating; water absorption after partial immersion in water for 24 h amounts to 0.013 kg/m^2^; water absorption by volume after complete water immersion for 28 days is equal to 0.96%. The material does not practically change its properties under conditions of long-term temperature alteration from −60 to +70 °C. The developed and implemented insulation systems for protective surfaces of framed construction objects, rubbhalls and frameless structures, floating floors, indoor ice rinks, and snow conservation systems are presented.

## 1. Introduction

Foamed polymers are widely used in insulating systems of the objects of various functional purposes. The reason for this is that the requirements for building constructions and engineering structures include standards for energy efficiency, durability, and functionality. The functionality of the insulating coating of any construction is associated with the creation of controlled temperature and humidity conditions in insulated structures and internal rooms [1,2]. Depending on the purpose of a particular object, the following requests may be in the forefront: control of temperature, humidity inside the premises, air freshness and purity, etc. In some cases (e.g., on ice arenas or by snow conservation), cold conservation is necessary [3,4].

Energy efficiency implies not only correspondence between the thermophysical properties of a structure and regulatory requirements, but also the stability of these properties over time. On the other hand, the durability of the structure is determined by the period of its maintenance-free exploitation, and consequently, by the operational durability of each element of this system [5,6].

The heat-insulating material, having a high-porous structure, enables the necessary thermal resistance of the construction and the stability of its properties over time. Thermal insulation protects the structural elements from temperature impacts, creating favorable conditions for maintenance-free exploitation of this structure. Being a “weak” element of the structure, heat-insulating material can deteriorate and lose its properties. Deterioration of the properties of the heat-insulating material in a construction can occur primarily as a result of mechanical, as well as temperature and heat-humidity impacts [7,8].

The durability of building structures depends extensively on the ability of heat-insulating materials, including foamed polymers, to resist the impact of alternating loads for a long time, as the subsequently occurred deformations and stresses have a significant effect on the structural frame of the foams and can cause their destruction.

Heat-insulating materials of two groups are used within the insulation systems of building constructions: with an operating temperature of up to 80 °C (these are mainly foamed polymers) and with an operating temperature exceeding 80 °C, which include products based on mineral wool, basalt and glass wool, foam glass, and expanded perlite [9,10].

Products based on polyolefin foams, polystyrene foam, polyurethane foam, polyisocyanurate foams, and other types of polymers are used within heat-insulating systems but in lower volumes. In terms of waterproofing properties, water resistance, and their thermotechnical characteristics, foamed polymers have better performance in comparison with mineral heat-insulating products (mineral wool slabs, or those made of foam glass); however, most of the foamed polymers are flammable, and this must be taken into consideration when choosing the field of exploitation or method of material installation [11,12,13].

Flammability appears to be the common feature of foamed plastics and their analogues. The development of measures aimed at reducing the fire hazard of constructions with application of foamed polymers, including reduction in combustibility of these materials, refers to the priority area of fundamental and applied research. As a result of the studies, two complementary directions have been developed: the injection of fire retardants (usually mineral powders) into the polymer composition and the application of chemicals that chemically react with the base material in order to obtain substances of reduced combustibility [14,15,16].

Insulation systems of building structures and household and construction objects are widely used and contribute significantly to heat and energy conservation. In terms of both thermal characteristics and permeability, it is necessary to achieve the correct effectuation of the joint between the slabs, regardless of the material of the slab products; however, the avoidance of heat loss and air infiltration is not always possible. Most insulation systems are based on the application of plate heat-insulating materials, whereas the joints between them and the junctions to the supporting structures represent heat transfer bridges. The concept of insulating coating developed by the authors implies the minimization of the number of such joints by means of the application of elastic foamed polymers and special methods of protecting the butt junctions [17,18,19].

The application of elastic products (mats, rolls) based on the polyethylene foam enables the realization of the proposed conception to the fullest extent. Considering the fact that polyethylene foam has low vapor and water permeability, the creation of seamless coating, above all, ensures protection against moisture and vapor–air mixture penetration through the insulating barrier.

In accordance with the technologies of OOO TEPOFOL, separate sheets are connected in a locking joint and welded with the hot air gun, subsequently forming a seamless insulating coating (patent of the Russian Federation, No. 2645190) [20,21]. Depending on the application conditions, the width of polyethylene foam rolls can reach up to 2 m, and the length of the material is made according to the customer’s size. An edge is formed on the sides of the rolls, which allows the creation of a lock joint.

**Figure 1:**
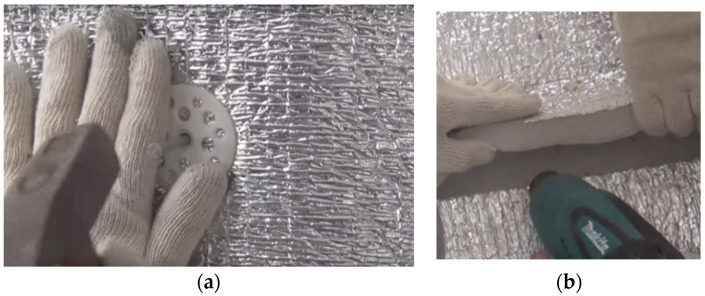
Installation of thermal insulation: (**a**)—mechanical fastening of the insulation sheet; (**b**)—welding of joints of polyethylene foam rolls.

The technology of formation of a seamless coating (Figure 1) is as follows. At the manufacturing stage, L-shaped lateral and end-type edges are formed on each sheet (roll) of polyethylene foam. At the mounting stage, the sheets (rolls) are mechanically fixed on the load-bearing structures, and the L-shaped edges are connected into a lock joint with subsequent welding with hot air gun, which allows quick and easy mounting of any structure. In order to create an insulating coating, it is also important to obtain a seamless joint of polyethylene foam sheets with the base of the structure, as well as connection between the insulation of the walls and ends of the construction. In case the welding joint is inadvisable, mechanical methods of fixation of the locking joint of the products are used.

**Figure 2:**
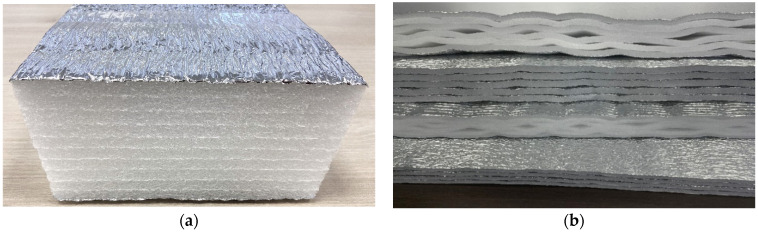
Polyethylene foam products: (**a**)—layered products; (**b**)—products of AirLayer line.

The classical technology of mounting involves the application of layered products with firm adherence of the layers (Figure 2). Products can be made with metallized coating (aluminum foil or metallized film) or without coating.

Expertise examination of usage of polyethylene foam products led to the development of a new line of materials—AirLayer (patent of the Russian Federation, No. 199048) [22]. These are heat-insulating multilayer materials containing flat layers of polyethylene foam (polypropylene or rubber), interconnected by means of seams forming air gaps between the layers. Layers can be made with or without metallized coating. Such a system has a lower thermal conductivity in comparison with multilayer materials, whereas insulating coatings demonstrate better thermal performance.

## 2. Method

In the presented studies, the properties of polyethylene foam samples were determined both under normal conditions and after climatic tests, and the fatigue characteristics of the material were evaluated.

Determination of the operating durability of polyethylene foam samples included the examination of the properties of the material under conditions of temperature and humidity impacts and the assessment of the creeping properties of the samples.

Assessment of the creeping properties of plastics under a dead load is used relatively often in the examination of their properties. The peculiarity of the method developed by NRU MGSU in collaboration with Vilnius Gediminas Technical University (VILNIUS TECH) is that the samples were preliminarily subjected to the climatic impact and then subsequently investigated for creeping [23,24,25].

Compressive creep of non-cross-linked polyethylene foam samples with and without a metallized coating (method GOST EN 1606-2011) was determined before climatic tests and on samples that have been climatically assessed.

Considering the properties of polyethylene foam, climatic tests were performed within 60 test cycles in two modes. Mode 1: temperature decrease to minus 20 °C—1 h; isothermal holding at minus 20 °C—1 h; temperature increase from minus 20 °C to +40 °C—1 h; isothermal holding at +40 °C—1 h; temperature decrease from +40 °C to minus 20 °C—1 h. Mode 2 simulated the conditions of the Arctic circle: temperature decrease to minus 60 °C—1 h; isothermal holding at minus 60 °C—1 h; temperature increase from minus 20 °C to +40 °C—1 h; isothermal holding at +40 °C—1 h; temperature decrease from +40 °C to minus 60 °C—1 h.

Compression strength of the polyethylene foam was determined within the interval of deformations from 0% to 12%. Products made of polyethylene foam with dimensions of 100 × 100 × 100 mm, 100 × 100 × 50 mm, 200 × 200 ×100 mm, and 200 × 200 × 50 mm with the ratio of area and thickness (geometric factor *S*/*h*, m^2^/m): respectively, 0.1; 0.2; 0.4; and 0.8 (m) have been tested.

Longitudinal tensile strength tests were performed in accordance with GOST EN 1608–2011. Metod opredeleniya prochnosti pri rastyazhenii parallel “no licevym poverhnostyam” (Thermal-insulation products used in construction. Method for determination of the tensile strength parallel to the front surfaces). Determination of moisture absorption and diffusion of the polyethylene foam was performed in accordance with GOST EH 12088-2011. Determination of water absorption ability of the samples (with coverage or without it) by partial submergence was performed in accordance with GOST EN 1609-2011. Thermal conductivity of the samples was determined in accordance with GOST7076-99.

Operating durability was determined by the property changes (strength, density, thermal conductivity) as a result of climatic impacts and within the process of long-term mechanical loads by the evaluation of creeping of the samples.

In order to assess the heat-protective qualities of the external wall made of a wooden frame insulated with a polyethylene foam sheet, an experimental determination of the heat transfer resistance on a selected section of the wall was performed in accordance with GOST 26254-84, «Zdaniya i sooruzheniya. Metody opredeleniya soprotivleniya teploperedache ograzhdayushchih konstrukcij» (Buildings and constructions.

**Figure 3:**
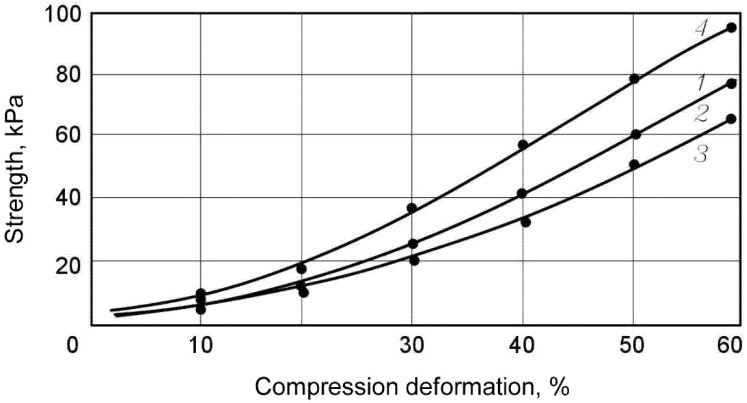
Deformation curves of the NXLPE samples. 1—control sample series; 2—samples subjected to the climatic impacts according to the Mode 1; 3—same with the Mode 2; 4—samples in the frozen state (at −60 °C).

Methods for determination of heat-transfer resistance of enclosing structures), GOST R 56623-2015. “Kontrol’ nerazrushayushchij. Metody opredeleniya soprotivleniya teploperedache ograzhdayushchih konstrukcij” (Non-intrusive inspections. Methods for determination of heat-transfer resistance of enclosing structures) and GOST 25380-2014 “Zdaniya i sooruzheniya. Metod izmereniya plotnosti teplovyh potokov, prohodyashchih cherez ograzhdayushchie konstrukcii” (Buildings and constructions. Method for determination of density of the heat flows permeating through the enclosing structures). The sensors were installed on both the internal and external wall surfaces (Figure 3). The results of experimental determinations of the resistance of the external wall made of a wooden frame with insulation of polyethylene foam sheet were: thermal resistance, 2.96 m^2^ °C/W, heat transfer resistance, 3.12 m^2^ °C/W.

## 3. Experiments and Results

The results of examination of the impacts of climatic factors on the alternations in physical and mechanical characteristics of the samples of non-cross-linked polyethylene foam (NXLPE) are shown in Figure 3 and in Table 1.

It has been established that natural deformations of polyethylene foam in the temperature range from −60 to +40 °C are insignificant. The state of the air–vapor mixture in the polyethylene foam cells changes under cooling from 0 to −60 °C. Water vapor transforms into a solid state and fine-crystalline ice appears on the internal surfaces of the cells at temperatures close to 0 °C. The gas pressure in the cells changes, and the compression of the material begins. The air density and the compression of the material increase with further temperature decrease.

**Figure 4:**
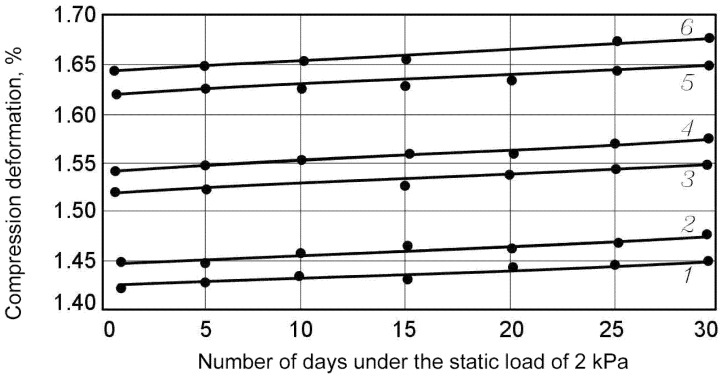
Compression deformation under the static load of 2 kPa depending on the number of days of loading. 1, 2—control samples; 3, 4—samples tested after the climatic impact according to Mode 1; 5, 6—samples tested after the climatic impact according to Mode 2; 1, 3, 5—foiled samples; 2, 4, 6—samples without foil.

**Figure 5:**
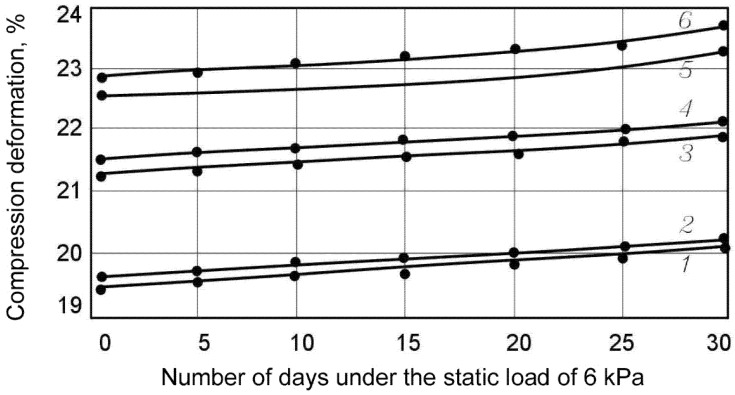
Compression deformation under the static load of 6 kPa depending on the number of days of loading. 1, 2—control samples; 3, 4—samples tested after the climatic impact according to Mode 1; 5, 6—samples tested after the climatic impact according to Mode 2; 1, 3, 5—foiled samples; 2, 4, 6—samples without foil.

Studies revealed that the material is compressed by 8–10% of its thickness under standard load (in accordance with the recommendations of GOST EN 1606-2011). Further tests showed that no deformation of the product under this type of load occurs (or they cannot be detected due to their insignificance). The effect of creeping absence was detected both for the control sample series and for the samples subjected to the climatic impacts according to both modes 1 and 2. For this reason, it was decided to increase the applied load up to 2–6 kPa. In this case, the alternation in the sample size was recorded by the sensors after the initial deformation; some of the results are presented in Figure 4 and Figure 5 and in Table 2.

The absence of deformations under standard loads and insignificant deformation under high loads allowed concluding that the products based on polyethylene foam are resistant to both climatic impacts and mechanical loading, which does not exceed 50% of the compressive strength at 10% deformation. No damage of the samples was revealed on completion of testing.

The results of climatic tests revealed that the decrease in the strength characteristics of polyethylene foam samples did not exceed 4–6%. The material has a high operating durability, which is equivalent to fifty years of exploitation in the temperature range from −60 to +60 °C. The use of products based on polyethylene foam is not desirable at temperatures above 80 °C, since the destruction of the polymer and significant plastic deformations of the product are possible.

Geometric factor impact (ratio of the area of the material sample to its thickness *S/h*) was estimated according to the change in the sample compression deformation (Table 3). It is explained by the deformation conditions of the sample under compression and the structure of the material. Polyethylene is an elastic material with high tensile strength. The pores of the polyethylene foam are filled with air, and the membranes (polyethylene) separating the cells are practically gas-impermeable, having very high tensile strength.

Research performed at the Research Institute of Building Physics, with the participation of the authors, revealed that thermal conductivity of the samples of the air layer line (layered products with air cavities) with an average density of polyethylene foam of 19–22 kg/m^3^ amounts to 0.029–0.038 W/(m∙K). Diffusion moisture absorption is equal to 0.44 kg/m^2^ without a metallized coating and 0.37 kg/m^2^ with a metallized coating; water absorption after partial immersion in water for 24 h amounts to 0.013 kg/m^2^; water absorption by volume after complete water immersion for 28 days is equal to 0.96%. The fracture pattern of the contact surface “polyethylene foam-metal” is cohesive along the adhesive layer, and the breaking stress was equal to 12–17 kPa.

Tests performed at the Research Institute have shown that the compressive strength at 10% deformation is determined by the function of load application area and varies from 70 kPa during the test of cube samples of 10 × 10 × 10 in size to 260 kPa for areas exceeding 100 m^2^. This fact makes it reasonable to use polyethylene foam for insulation of large flat surfaces: for example, flat roofs under reinforced screed or floating floors.

Products made of polyethylene foam can be used under conditions of negative (from minus 60 °C) and alternating temperatures. It has been established that the change of properties of polyethylene foam in the considered temperature range (from −60 to +40 °C) does not exceed the experimental error. It was found that, at a temperature of −60 °C, the shrinkage of polyethylene foam does not exceed 1.5%. Shrinkage can be explained by the change in the air density in the polyethylene foam cells and the creation of a partial vacuum. By the insulation of the objects of considerable length, the temperature changes in the material must be taken into account when designing joints and fastenings of insulating elements.

**Figure 6:**
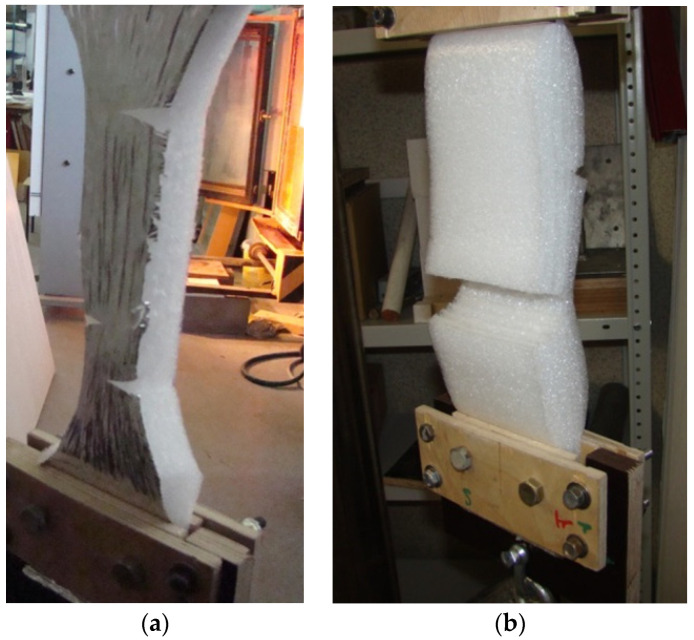
Tests for determination of the ultimate tensile strength of polyethylene foam samples in the longitudinal direction: (**a**)—for a seamless product; (**b**)—along the welding seam.

The elastic insulating coating is deformed together with the base on which it is mechanically fixed. This base is usually represented by a wooden or metal frame. Temperature and humidity (by the wood) deformations appear in the construction depending on the parameters of the external environment. These deformations imply the occurrence of tensile stresses in the insulation coating. It is important to preserve the properties of the insulating coating (and, first of all, its integrity) in order to ensure the durability of the construction in general. The experimental tests (Figure 6) revealed that the ultimate longitudinal tensile strength along the blanket with a metallized coating is 80–92 kPa; without metallized coating, it is 80–87 kPa; and the ultimate longitudinal tensile strength of the weld seam amounts to 29–32 kPa.

It has been established that the values of thermal conductivity of polyethylene foam with an average density of 18–20 kg/m^3^ amounts to 0.032–0.034 W/(m·K); diffusion moisture absorption is equal to 0.44 kg/m^2^ without a metallized coating and 0.37 kg/m^2^ with a metallized coating; water absorption after partial immersion in water for 24 h amounts to 0.013 kg/m^2^; and water absorption by volume after complete water immersion for 28 days is equal to 0.96%.

It has been established that the presence (absence) of the coating on the products (in this case, metallized lavsan) and climatic impacts have practically no effect on the mechanical properties of polyethylene foam samples. Increases in the strength by means of the coating do not exceed 2.1%, which is less than the statistical error of the experiment, equal to 4.9%.

The goals of the field study were as follows: assessment of the humidity state of wood and insulation—polyethylene foam—as well as of the heat-shielding properties of the outer wall. The external wall of the house is sheathed with the plywood of 10 mm in thickness along the polyethylene foam with subsequent cladding with plastic siding.

The outdoor air temperature was minus (12 ± 2) °C, relative humidity was 80%, and water vapor content (absolute humidity) was 1.7 g/m^3^. The outdoor air temperature was plus (23 ± 2) °C, relative humidity was 20%, and water vapor content (absolute humidity) was 4.7 g/m^3^. The framed structure of the exterior walls of the examined house consists of a pine bar 150 × 150 mm, the space between which is filled with pieces of different-sized polyethylene foam; from the outside, the frame is wrapped with a joined sheet of TEPOFOL^®^ foam of 50 mm in thickness with a metallized coating. The joints of the canvas are welded with a hot air gun in accordance with the manufacturer’s technology.

**Figure 7:**
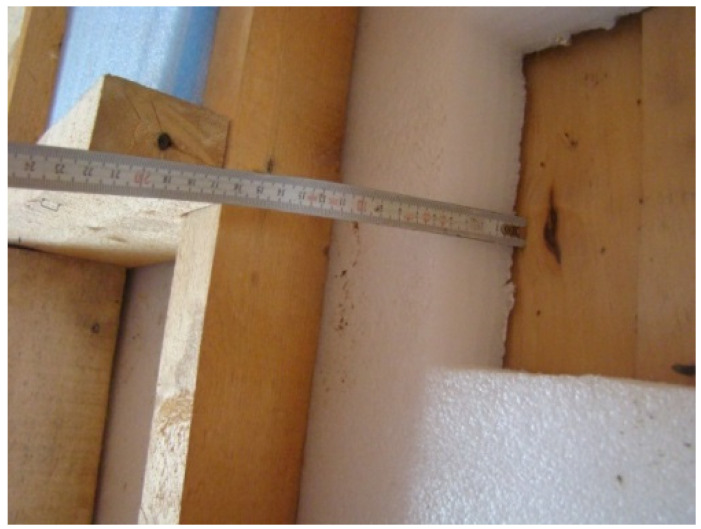
Place where the samples and fragments of pine timber in the outer wall of the building were taken.

**Figure 8:**
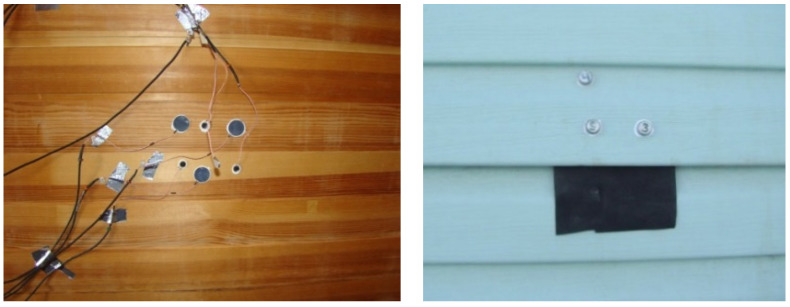
Installation of the sensors of temperature and heat flows.

The moisture measurements of the pine lining (interior decoration) were performed with the help of an express-measurer of humidity and thermal conductivity, IVTP-12, in accordance with GOST 8.621-2006. Average values of moisture by weight valued about 14% wt. In order to assess the moisture condition of the construction materials that represented the external wall of the building, polyethylene foam samples and a fragment of a pine log were taken (Figure 7 and Figure 8). The samples were placed in sealed bags and transported to the laboratory for thermogravimetric moisture determination.

Within the process of layer-by-layer dismantling of the materials of the external wall, the wetting zones of the internal surface of the polyethylene foam sheet, which enveloped the frame of the house, were revealed by visual inspection. Moisture was found in the areas where the insulant in the frame consisted of separated pieces and where it was laid with marked seams and loose-fitting adjunction. No mold or rot were detected on the timber frame elements.

## 4. Discussions

Insulation systems with the application of polyethylene foam make it possible to solve a complex of tasks that, to one degree or another, ensure the energy efficiency of building, housing, and special objects. The implementation of the concept of seamless insulation coatings enables realization of the following energy efficiency components.

First of all, it is the elimination of direct heat losses through the insulating coating or cold preservation in the insulated object. Secondly, it is the protection of structures from external atmospheric impacts or internal aggressive environments that contributes to providing the durability of these structures. Thirdly, it makes it possible to build an optimal internal microclimate, depending on the functional purpose of the object.

The economy segments where the systems of seamless insulation made of polyethylene foam are used represent residential and industrial constructions, erection of agricultural facilities, insulation of sports facilities, and such an innovative area as snow conservation [26].

**Figure 9:**
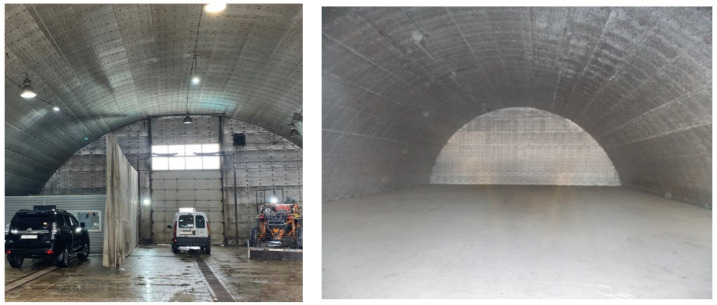
Insulation systems of frameless objects.

Insulation systems with the application of polyethylene foam with a seamless joint showed good results in the insulation of garages, warehouses of agricultural products, and livestock facilities (Figure 9). In the considered systems, it is possible to distinguish properties both typical of all types of objects and particular ones, which should be necessarily implemented in order to solve specific operational problems.

By insulation of frameless structures, polyethylene foam rolls are laid parallel to the base of the hangar. In areas of mechanical fastening, additional copper-plated nails (pins) are welded at a distance of 100–120 mm from each other along the locking joint, and at a distance of 100–150 mm from the center of the locking joint. Polyethylene foam is mounted to the bearing surface by pinning on welded pins and fixed with pressure washers. The locking joints of the material must be necessarily soldered with a construction (industrial) hot air gun in order to achieve the “splicing” of the insulating sheets.

The common goal lies in the preservation of the internal environment of the object at an established, normalized level while optimizing energy consumption. In this case, the main advantages of the seamless coating become apparent: its thermal, vapor, and water insulating properties, as well as high durability. Under conditions of negative external temperatures, the coating contributes to heat consumption minimization; in a hot climate, it contributes to a reduction in the expenditures for air conditioning, supply, and exhaust ventilation.

By insulation of hangars designed for covered parking lots, the goals of protective measures are as follows: maintaining of equipment in working state, creating favorable working conditions for personnel, saving energy, and reduction in expenditures for both facilities and equipment exploitation. From engineering systems, air exchange systems and modern powerful forced ventilation systems can be recommended. In the premises designed for storing the vegetable products, temperature and humidity control should be necessarily performed, which will ensure maximum preservation of products.

**Figure 10:**
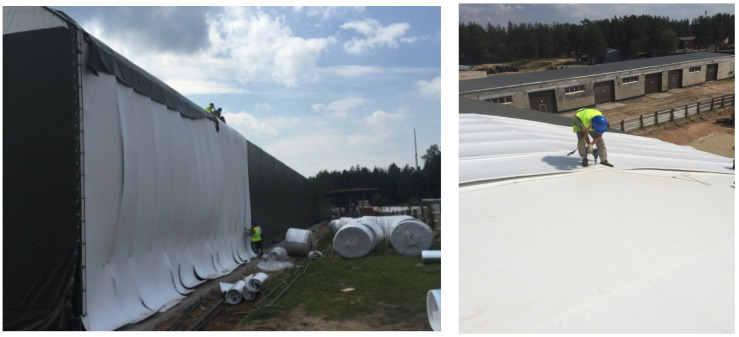
Installation of a tent covering over a layer of polyethylene foam roll.

Tent structures, which are of framed type as a rule (Figure 10), are used in the erection of long span constructions: sports facilities, storage facilities, and warehouses. Systems made of profiled metal and light metal structures are used in the quality of a frame.

Mounting of insulation system intended for rubbhall (primarily for insulation and protection against condensed moisture) is performed in the following sequence. A lath is installed on the load-bearing frame; polyethylene foam roll is laid out on the lath; the sheets are fixed mechanically; a locking joint of sheets is formed; and the welding is performed. Next, the tent canvas is stretched and mechanically fixed along the perimeter and stiffening ribs of the structure. Such tent coverage protects the system well from all types of atmospheric impacts, but it cannot provide protection from unauthorized entry. For this reason, rubbhalls are recommended to be installed in protected areas.

**Figure 11:**
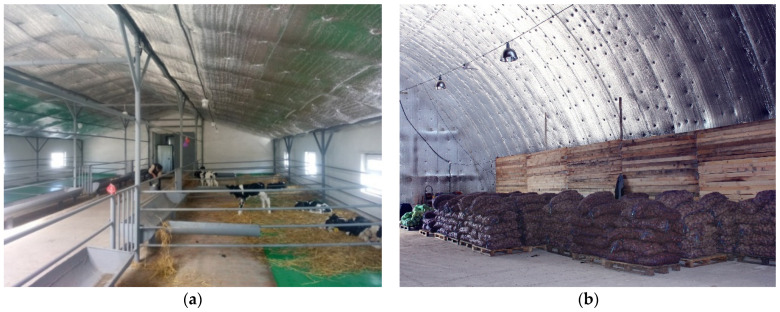
Insulation of agricultural facilities: (**a**)—covered stockyard; (**b**)—storage.

Insulation of framed systems can be performed both along the internal and external contour of the building relative to the load-bearing structure. The building can be intended for residential usage as well as for utility or agricultural needs (Figure 11).

**Figure 12:**
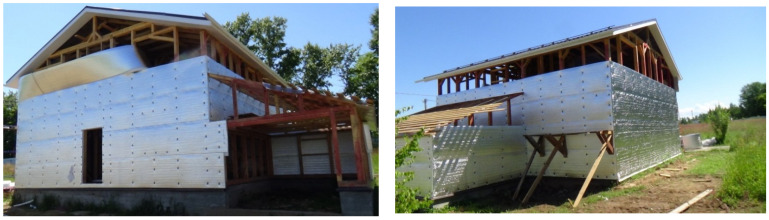
Installation of polyethylene foam sheet in a framed system.

In low-rise housing construction, the insulation of framed system is performed along the external contour (Figure 12). Polyethylene foam rolls are unfolded around the perimeter of the building and fixed to the timber posts with cap screws. The rolls on the contact surfaces are connected in a butt-joint and welded with hot air. Fixation of polyethylene foam sheet in a framed system is performed in the same way.

Considering that the insulating coating is located along the outline of the framed system, the humidity state of the frame becomes the most important issue. The field study accomplished by the specialists of the Research Institute of Building Physics, aimed at the following: assessment of the humidity state of wood and insulation—polyethylene foam—as well as of the heat-shielding properties of the outer wall. Moisture was found in the areas where the insulant in the frame consisted of separated pieces and was laid with marked seams and loose-fitting adjunction. No mold or rot were detected on the timber frame elements.

Thermal insulation made of polyethylene foam does not decompose or rot, which, in this case, is the advantage of the material, as such an insulating coating will serve throughout the entire service life of the structure without replacement.

In order to assess the heat-protective qualities of the external wall made of a wooden frame insulated with a polyethylene foam sheet, an experimental determination of the heat transfer resistance was performed on a selected section of the wall, which amounted to 2.96 W/m^2^ °C. If standards of air exchange and air conditioning requirements are respected and the internal micro-climate is maintained according to regulatory demands, conditions for systematic moisture accumulation and wood dampening do not occur.

The efficiency of seamless insulation coating made of polyethylene foam is so high that it is possible to build houses with energy consumption approaching zero, if energy-efficient glazing and accompanying architectural and engineering solutions (air recuperation, etc.) are used.

The performed research and calculations made it possible to conclude that the strength characteristics of polyethylene foam “Tepofol” allow using this material in floating floor systems in the quality of thermal insulation, and its low vapor permeability and water absorption enable its simultaneous usage as a water and vapor barrier.

A heat-insulating layer of polyethylene foam roll of required thickness is laid on a solid base. The insulant is tightly pressed against the wall of the load-bearing structure with the side thrust and is attached to the solid base of the floor at a distance of up to 150 mm from the walls of the load-bearing structure along the entire perimeter. After layering of thermal insulation, the locking systems located at the joints of the rolls are welded together (under thermal influence) in a way that an integral sealed thermal insulation canvas is obtained.

**Figure 13:**
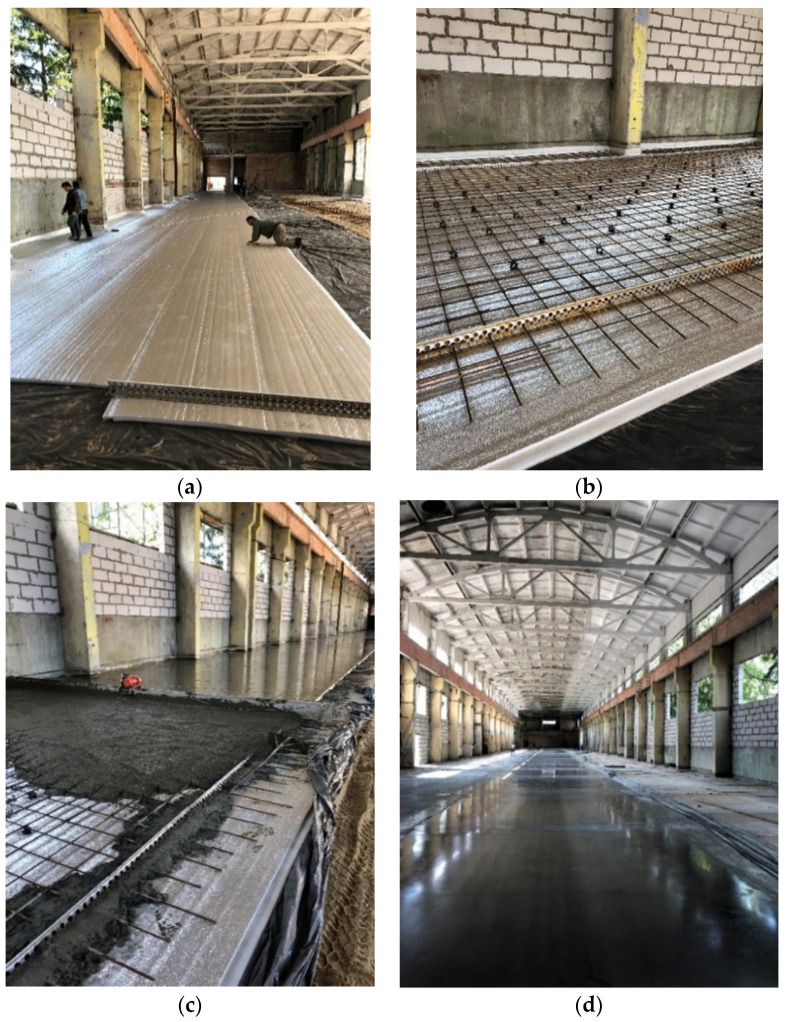
Floating floor installation: (**a**)—laying and welding of heat-insulating rolls; (**b**)—reinforcement; (**c**)—concrete laying; (**d**)—finished floor.

**Figure 14:**
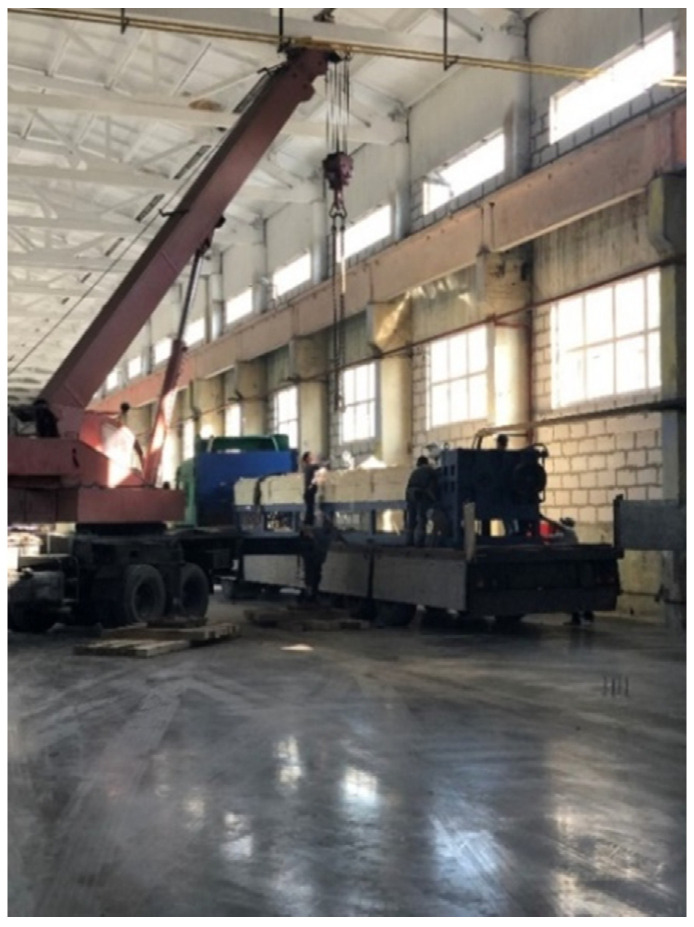
Installation of technological equipment by means of a crane.

The next stage is the installation of reinforcement cages, dividing elements, and additional reinforcement of the base in the places where the manufacturing equipment is supposed to be located; after that, the concrete laying with subsequent compaction of its surface by vibration is performed. After curing, the floor is topped and troweled, and a final protective coating is applied (Figure 13). The installation of the technological equipment becomes possible after 28 days of hardening (Figure 14).

The performance characteristics of polyethylene foam, as well as insulating coatings based on it, comply thoroughly with the requirements for materials and systems operating under the load, in contact with a wet base, as well as at negative and alternating temperatures. From the point of view of maintenance of temperature conditions in the insulating volume, the best way is to build a seamless joint as a result of the locking connection of separate polyethylene foam rolls using thermal welding. This technology also contributes to a significant increase in the heat-protective properties of the insulating coating by means of cold bridges minimization and elimination of leaks in the connection of separate insulating elements and on the surfaces of abutment to structures.

Monitoring of the insulating coating condition of the floating floor was performed when the communications were taken beyond the workshop with an underground laying. Studies revealed that the compression deformation of the polyethylene foam inside the insulating coating did not exceed 1%, the properties of the polyethylene foam did not change, and no abruption or other destruction of the insulating coating was detected.

**Figure 15:**
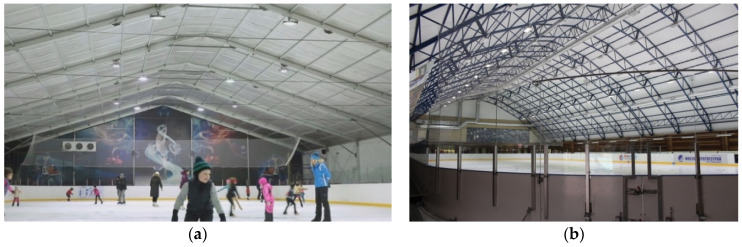
Insulation systems of covered ice rink (**a**) and ice arena (**b**).

Planification and construction of structures with spaces covered with artificial ice (Figure 15) represents a rather challenging task. Nowadays, modern ice rinks have two built-in pipe systems: cooling pipes and heating systems. Supply and exhaust ventilation providing fresh air supply is also obligatory. The insulating coating should imply the following tasks: firstly, the creation of comfortable conditions for users; and secondly, the reduction in energy expenditures for maintaining the ice in a working state by means of heat loss elimination through the structural elements.

The “conservation” of snow in the off-season (from spring to autumn) an original and commercially reasonable solution for the purpose of its early usage at ski resorts or in order to form snow reserves to ensure (compensate) its deficit in case of insufficient natural fallout in winter on skiing pistes of any type.

Polyethylene foam rolls are prepared and laid on the insulating surface. The rolls are connected in a lock joint according to the TEPOFOL technology. This is the stage when a specially developed technology was applied—the so-called “thermal blanket”.

**Figure 16:**
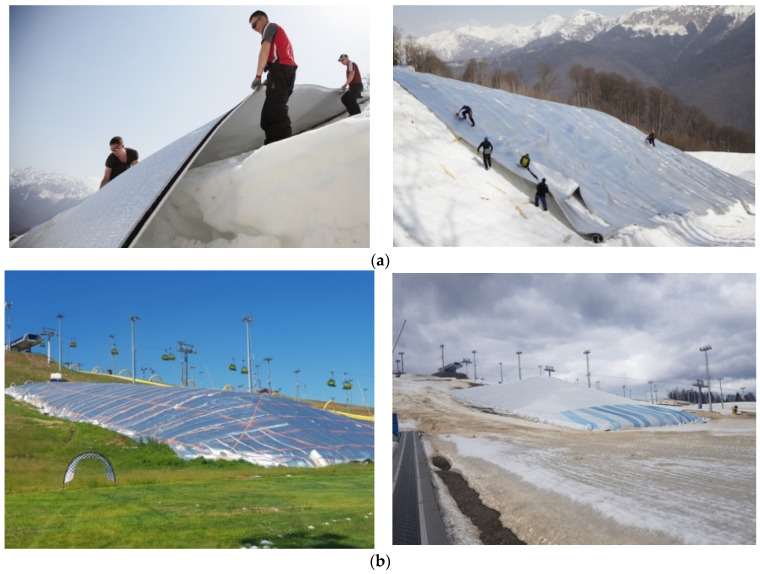
Mounting of a protective coating with the help of polyethylene foam rolls (snow conservation) and its exploitation: (**a**)—laying of the rolls; (**b**)—stored snow reserves under the “thermal blanket”.

Conservation of snow banks is performed with application of polyethylene foam roll of 20 mm in thickness with a metallized coating (Figure 16). In order to expand the durability of insulation system and enable its repeated usage, the arrangement of a tent is recommended, which is fixed along the perimeter of the snow storage and surface area with the help of tension structures: ropes are also fastened along the perimeter and represent a net.

From the point of view of thermophysics and heat-exchanging processes, the following phenomena are in evidence in this case. First and foremost, polyethylene foam has a low thermal conductivity, vapor permeability, and water absorption, and its properties do not practically change in the considered operating conditions. Secondly, the locking junction of the rolls allows minimizing the bridges of heat transfer at the joints and creates a seamless insulating coating. Thirdly, the snow itself has a relatively high heat capacity (of about 2080–2100 J/(kg∙°C) and relatively low thermal conductivity. Where the density of soft snow is 200 kg/m^3^, its thermal conductivity is equal to 0.15 W/(m·K), and the density of compacted snow amounts 600–700 kg/m^3^ by thermal conductivity—0.6–0.7 W/(m·K).

The metallized surface of the rolled polyethylene foam reflects the sun rays and as for the tented covering—it is also recommended to choose it in light tones in order to achieve sun rays’ reflection as well. The specified factors allow obtaining the thermal resistance of the insulating coating (“thermal blanket”) of about 1.3 W/m^2^ °C, if taking the thermal resistance of the snow mass into account.

Taking all the functional features of the realization of insulation systems for snow conservation into consideration, the principles of protection and preservation (conservation) of the snow coverage were developed and implemented on the mountainside of the Olympic resort Rosa Khutor in Sochi within the period 2013–2014 and on the eve of the 2014 Winter Olympics. By the beginning of the 2014 Winter Olympics, it was the only way to accumulate about half a billion cubic meters of snow mass here.

## 5. Conclusions

Polyethylene foam is one of the most representative examples of the application of high-molecular polymers obtained as a result of fundamental research in such applied areas as construction, tourism, etc.

Polyethylene foam and products based on it enable creating the effective systems of thermal insulation, as well as water and vapor barrier of the objects used in almost all areas of the building complex and engineering systems operating at temperatures up to 80 °C under conditions of the implementation of a seamless insulating coating.

The efficiency of seamless insulation coating made of polyethylene foam is so high that it is possible to build houses with energy consumption approaching zero, if energy-efficient glazing and accompanying architectural and engineering solutions (air recuperation, etc.) are used. “AirLayer” materials have lower thermal conductivity in comparison with multilayer materials, whereas insulating coatings demonstrate better thermal performance.

Systems with application of polyethylene foam have been tested in low-rise cottage construction by insulation of framed and frameless buildings and structures, in floating floor systems, and on flat and pitched roofs. The technology of cold conservation is implemented within the scope of construction of specialized warehouses and storage facilities by the insulation of indoor sports facilities as well as in the area of snow conservation.

Namely, foamed polymers differ from other types of heat-insulating materials in a whole range of valuable properties that allow the building of an effective insulating coating of building structures. Such properties of polyethylene foam as high elasticity and weldability at low temperatures allow creating seamless insulating coatings and make this material stand out in comparison with other types of thermal insulation. Revealing the abilities and advantages of polyethylene foam in the quality of an insulating multifunctional material contributes to the expansion of the scope of application of foamed plastics, in particular and polymers in general.

## Figures and Tables

**Table 1:** Strength of NXLPE samples at various compression deformations.

Samples Series		Compression (kPa) Deformation Strength, %					
		10	20	30	40	50	60
Control series	without foil	4	6	22	33	56	79
	with foil	4	6	23	34	56	78
Tests after the climatic impact according to Mode 1 (from −20 to +40 °C)	without foil	4	6	21	33	54	77
	with foil	4	6	22	34	55	78
Tests after the climatic impact according to Mode 2 (from −60 to +40 °C)	without foil	4	6	20	30	50	70
	with foil	4	6	21	32	53	75
Tests by frozen state at −60 °C	without foil	7	20	30	50	75	92
	with foil	8	21	32	53	79	96

**Table 2:** Results of Deformation Strength test under the static load.

Samples Series		Deformation Strength (%) with the Number of Days of Loading (τ) under the Static Load (σ, kPa)					
		σ = 2 kPa		σ = 4 kPa		σ = 6 kPa	
		τ = 5	τ = 30	τ = 5	τ = 30	τ = 5	τ = 30
Control series	without foil	1.5	1.5	9.8	10.0	19.7	20.5
	with foil	1.4	1.4	9.7	9.9	19.6	20.1
Tests after the climatic impact according to Mode 1	without foil	1.6	1.6	10.1	10.3	21.5	22.1
	with foil	1.5	1.5	10.0	10.2	21.3	21.9
Tests after the climatic impact according to Mode 2	without foil	1.7	1.7	10.4	10.7	22.9	23.6

**Table 3:** Dependency of the compressive strength of polyethylene foam samples on compression deformation and geometric factor.

Compression Deformation, %	Geometric Factor *S*/*h*, m^2^/m			
	0.1	0.2	0.4	0.8
2	10	20	32	50
10	68	81	170	260

## Data Availability

Research data is available in the databases ORCID, Scopus, WoS Core Collection, VoS RSCI.
